# Provider perspectives on clinical decision support to improve HIV prevention in pediatric primary care: a multiple methods study

**DOI:** 10.1186/s43058-023-00394-7

**Published:** 2023-02-21

**Authors:** Julia Pickel, Alexander G. Fiks, Dean Karavite, Pegah Maleki, Rinad S. Beidas, Nadia Dowshen, Danielle Petsis, Robert Gross, Sarah M. Wood

**Affiliations:** 1 Wake Forest University School of Medicine, Winston-Salem, NC USA; 2 Clinical Futures and the Department of Pediatrics, Children’s Hospital of Philadelphia, Philadelphia, PA USA; 3 Department of Biomedical and Health Informatics, Children’s Hospital of Philadelphia, Philadelphia, PA USA; 4 Department of Oncology, University Pennsylvania Perelman School of Medicine, Philadelphia, PA USA; 5 Department of Medical Social Sciences, Feinberg School of Medicine, Northwestern University, Chicago, IL USA; 6 Division of Adolescent Medicine, Children’s Hospital of Philadelphia, Philadelphia, PA USA; 7 Department of Pediatrics, Perelman School of Medicine, University of Pennsylvania, Philadelphia, PA USA; 8 Department of Medicine (Infectious Diseases), University of Pennsylvania Perelman School of Medicine, Philadelphia, PA USA; 9 Center for Clinical Epidemiology and Biostatistics, University of Pennsylvania Perelman School of Medicine, Philadelphia, PA USA

**Keywords:** Pediatrics, Primary care, Implementation science, HIV, Pre-exposure prophylaxis, Implicit bias

## Abstract

**Background:**

Clinical decision support (CDS) is a promising intervention for improving uptake of HIV testing and pre-exposure prophylaxis (PrEP). However, little is known regarding provider perspectives on acceptability, appropriateness, and feasibility of CDS for HIV prevention in pediatric primary care, a key implementation setting.

**Methods:**

This was a cross-sectional multiple methods study utilizing surveys and in-depth interviews with pediatricians to assess acceptability, appropriateness, and feasibility of CDS for HIV prevention, as well as to identify contextual barriers and facilitators to CDS. Qualitative analysis utilized work domain analysis and a deductive coding approach grounded in the Consolidated Framework of Implementation Research. Quantitative and qualitative data were merged to develop an Implementation Research Logic Model to conceptualize implementation determinants, strategies, mechanisms, and outcomes of potential CDS use.

**Results:**

Participants (*n* = 26) were primarily white (92%), female (88%), and physicians (73%). Using CDS to improve HIV testing and PrEP delivery was perceived as highly acceptable (median score 5), IQR [4–5]), appropriate (5, IQR [4–5]), and feasible (4, IQR [3.75–4.75]) using a 5-point Likert scale. Providers identified confidentiality and time constraints as two key barriers to HIV prevention care spanning every workflow step. With respect to desired CDS features, providers sought interventions that were integrated into the primary care workflow, standardized to promote universal testing yet adaptable to the level of a patient’s HIV risk, and addressed providers’ knowledge gaps and bolstered self-efficacy in providing HIV prevention services.

**Conclusions:**

This multiple methods study indicates that clinical decision support in the pediatric primary care setting may be an acceptable, feasible, and appropriate intervention for improving the reach and equitable delivery of HIV screening and PrEP services. Design considerations for CDS in this setting should include deploying CDS interventions early in the visit workflow and prioritizing standardized but flexible designs.

**Supplementary Information:**

The online version contains supplementary material available at 10.1186/s43058-023-00394-7.

Contributions to the literature

- Although prior implementation science research has addressed barriers to HIV prevention services in primary care, our focus on the pediatric setting, where provider self-efficacy for HIV prevention is lower, is unique.
- We utilized an integrated multiple methods approach from both implementation science and clinical informatics to assess not only what barriers exist to implementation, but also where they may arise in the clinical workflow.
- Our paper is the first to present an implementation research logic model for utilizing clinical decision support as an intervention to improve HIV prevention in the pediatric primary care setting.

## Background

Despite clinical practice guidelines, HIV testing and pre-exposure prophylaxis (PrEP) remain underused in pediatric primary care. HIV testing is a critical adolescent health screening, as adolescents account for 20% of U.S. incident HIV diagnoses. Early HIV diagnosis and viral suppression with treatment dramatically lower morbidity and mortality and prevent secondary transmission. For at-risk youth who test negative for HIV, PrEP is > 90% effective in preventing HIV [1, 2]. Previous analyses have identified low HIV testing rates both relative to the overall prevalence of sexual activity and sexually transmitted infections (STI) in adolescents [3, 4]. Despite high efficacy, PrEP delivery and counseling rates also remain low, with only 16% of youth ages 16–24 with PrEP indications receiving prescriptions [2, 5–7] despite Centers for Disease Control and Prevention (CDC) guidelines now recommending all sexually active adolescents should be informed about PrEP [8].

Pediatric primary care, where most adolescents receive health services, is an ideal setting for HIV testing and PrEP delivery. However, tight schedules and competing patient needs often lead to increased provider mental workload and decreased situational awareness—an impaired ability to recognize and synthesize patient risk factors and efficiently identify the need for care [9, 10]—regarding HIV risk. In busy clinical settings, cognitive tunneling, a process by which individuals consider fewer solutions, ideas, and cues due to an inability to manage excess information, impairs decision-making [9]. Low situational awareness in primary care can thus result in providers failing to recognize key opportunities for HIV prevention, for example, identifying the need for HIV testing for adolescents with STIs given their increased biologic and social risk [11]. Prior research demonstrates that pediatricians are often hesitant to ask about sexual behavior, sexual orientation, and gender identity and concerned about confidentiality of this information. These essential data points are often absent in pediatric health records, although an informative history can both lead to improved delivery of HIV prevention services and cultural tailoring of services to key populations, such as young men who have sex with men (MSM) or transgender youth [12]. This discomfort around sexual health delivery may result in lower prioritization of HIV and STI prevention within the multitude of preventive health topics addressed during the pediatric visit [13].

Increased mental workload also heightens implicit bias, wherein unintentionally held attitudes and beliefs affect clinical decision-making [10, 14, 15]. Inequities by gender, sexual orientation, race, sex, and socioeconomic status [16–19] exist at nearly every step of PrEP delivery, from counseling to prescription, with provider implicit bias playing a key role [20]. Previous research demonstrates lower rates of PrEP counseling and prescription for Black men compared to White men and for cisgender women who have sex with men compared to cisgender MSM [21]. While women ages 15–24 years old account for the vast majority of STIs [22, 23], PrEP initiation rates are substantially lower in cisgender heterosexual females compared to cisgender MSM [7, 21, 23, 24]. Data demonstrate that 10% of cisgender females with PrEP indications have been prescribed PrEP compared to 28% of cisgender males [25]. These inequities may be further magnified in adolescents, with a recent study of adolescents with STIs finding that male sex at birth patients were 26 times more likely than female sex at birth patients to receive PrEP counseling [5]. Within the pediatric primary care setting, given overall low rates of testing, there is thus a critical need for guideline-based HIV prevention service delivery to youth across sexes, sexual orientations, and gender identities. Given these challenges, developing methods for providers to manage information and efficiently apply relevant guidelines is a critical step to improve HIV testing and PrEP delivery in pediatric primary care.

Clinical decision support (CDS) for HIV prevention aims to aid provider decision-making, increase adherence to HIV testing and PrEP guidelines, decrease bias in guideline application, and improve efficiency and effectiveness of care delivery [9, 26]. Clinical decision support encompasses “knowledge-driven interventions that can promote safety, education, workflow improvement, communication, and improved quality of care” [27]. Electronic and paper alerts and reminders, order sets, and guidelines are all types of CDS, providing a prompt toward evidence-based decision-making and away from biased clinical decisions. However, to be successfully implemented, CDS must be carefully designed to fit within existing workflows and users’ cognitive processes [9, 28]. The application of user-centered design in the development of CDS for primary care can help facilitate better CDS implementation and utilization [29].

The primary objective of this multiple methods pre-implementation study was to understand perceptions of the acceptability, appropriateness, and feasibility of electronic health record (EHR)-based CDS as an intervention to improve the equity of HIV testing and PrEP delivery in pediatric primary care. Secondarily, we aimed to identify optimal timing and describe context-specific barriers and facilitators of CDS implementation. Lastly, we sought to integrate these data to develop an Implementation Research Logic Model characterizing determinants, strategies, mechanisms, and outcomes for future implementation of CDS to improve HIV testing and PrEP delivery in pediatric primary care.

## Methods

### Design, setting, and participants

This cross-sectional concurrent multiple methods (QUAL + quan) study [30] recruited providers from four urban primary care clinics within a large pediatric academic health system based in Philadelphia, PA, USA. The clinics see > 100,000 visits annually. Over half of the families live at or below the poverty line, > 69% have Medical Assistance coverage, and ~ 70% are African American [31]. Two of the clinics provide government-subsidized (Title X) family planning services. None offer HIV point-of-care testing, and three have in-clinic phlebotomy. Eligible participants were nurse practitioners, physician assistants, or pediatric physicians. Participants were recruited via emails sent by their practice directors describing the study’s aim and providing contact information for the Principal Investigator. Those opting to participate submitted a brief online form, all of whom completed study visits. We targeted a sample size of 20–25—generally thought to be sufficient to achieve saturation of themes in qualitative research [32]. Recruitment ended when the qualitative coding team confirmed that saturation was met (i.e. no new themes were arising from the qualitative data). We utilized the Consolidated Criteria for Reporting Qualitative Research (COREQ) reporting guidelines (see Additional File 1).

### Study procedures

Our multiple methods approach utilized separate quantitative and qualitative methods to answer distinct subdomains of the larger question of how CDS might best improve HIV prevention service delivery [30]. Participants completed a single visit consisting of computer-assisted survey instruments assessing attitudes toward CDS and considerations for designing effective CDS. These measures were followed by individual semi-structured interviews eliciting HIV prevention-related steps in the primary care workflow, exploring barriers and facilitators to CDS implementation, and discussing how CDS might address inequities in HIV screening for cisgender females in particular, and across diverse populations of youth [6]. Interviews were conducted by a female research assistant holding a BA degree (J.P.) who received qualitative interview training from two researchers holding MD, MSHP (S.W.), and MPH (D.P.) degrees. Participants had no prior relationship with the interviewer. Audio-recorded interviews were conducted in providers’ clinical offices and lasted 25–45 min. No additional written fieldnotes were recorded. Participants were compensated $25.

### Quantitative measures

#### Demographics

Surveys assessed provider age, race, ethnicity, sex, gender, clinic, educational degree, formal adolescent medicine training, and years in practice.

#### Provider characteristics

Participants were asked how many adolescents they saw on average monthly. Frequency of discussing sexual activity with their patients was measured on a 5-point Likert scale. Responses were categorized as infrequently/never, about half the time, and most/all of the time. Providers were asked if they had heard of PrEP prior to the study and how many times they prescribed or referred to another clinic for PrEP.

#### Acceptability, appropriateness, and feasibility of CDS

The perceived acceptability, appropriateness, and feasibility (key measures in the Proctor implementation outcomes framework [33]) of implementing EHR-based CDS to improve HIV testing and PrEP delivery were measured using the Acceptability of Intervention Measure (AIM), Intervention Appropriateness Measure (IAM), and Feasibility of Intervention Measure (FIM) [34], validated Likert scale measures with a maximum score of 5 (“Completely agree”).

#### Design considerations for CDS

Participants ranked a set of seven proposed CDS tools in terms of appeal from 1 (highest) to 7 (lowest). These tools were based on existing primary care system EHR tools for asthma and immunizations that had demonstrated measurable improvements in patient outcomes [35–39]. The proposed tools included an (1) electronic self-screening questionnaire capturing sexual activity information in the EHR, (2) automated referrals to prevention counselors triggered by STI results, (3) HIV testing alerts triggered by STI results, (4) HIV testing alerts for all adolescents, (5) default HIV test orders, (6) a bundled tool including alerts and defaulted STI and HIV test orders and (7) PrEP counseling alerts triggered by STI results. 

### Quantitative analysis

We used summary statistics to analyze survey findings, using Stata 15 (StataCorp, College Station, TX). Median and interquartile ranges (IQRs) were used for items with a non-normal distribution.

### Qualitative measures

The first part of the interviews used a hypothetical clinical case vignette of an adolescent with a new STI to elicit the HIV prevention workflow in primary care and explore context-specific barriers and facilitators to HIV testing and PrEP initiation. Each vignette was pilot tested with five pediatricians prior to use. Vignettes were chosen at random for each participant (Supplementary Fig. 1). The second part of the interview, informed a priori by the Consolidated Framework for Implementation Research (CFIR), asked questions regarding multi-dimensional barriers and facilitators to CDS as an intervention [40]. We focused on four CFIR constructs: (I) Intervention Characteristics (e.g. cost, adaptability, trialability), (II) Inner Setting (clinic context), (III) Outer Setting (external/environmental considerations and patient needs), and (IV) Characteristics of Individuals (primary care providers). We omitted the Process construct given the study’s pre-implementation focus.

### Qualitative analysis

Interviews were transcribed by an independent agency and coded using two approaches to understand (1) the discrete steps of the primary care workflow where CDS might influence provision of HIV testing or PrEP services, and (2) multi-level barriers to CDS implementation. Transcripts were coded by three members of the research team (D.P., P.M., J.P.). We first performed a work domain analysis, a method which evaluates the purposes, values, functions, and resources of a given work system [41–43]. We used responses to the vignettes to delineate the workflow and decision-making steps faced in primary care encounters, aiming to understand how each workflow step could be a locus for future CDS intervention. We added each workflow step to our codebook, and text relating to each step was thematically analyzed to identify barriers and facilitators to CDS delivery. Secondly, we used a deductive approach to map barriers and facilitators to using CDS to CFIR domains. Within the CFIR domains, interview text was coded to identify salient themes. To achieve inter-rater reliability for both coding processes, transcripts were independently coded in Nvivo 12 Plus (QSR International Pty Ltd. Version 12, 2018) by two raters until the kappa statistic for each coding team reached 0.9. Discrepancies were resolved through consensus.

### Data integration

We used quantitative findings from the ranking exercises, key findings from the work domain analysis, and themes which emerged from our CFIR coding to develop an Implementation Research Logic Model (IRLM). The IRLM approach aims to describe the determinants, strategies, mechanism, and outcome processes that may improve the adoption of interventions in health care [44]. We utilized three distinct implementation science frameworks to comprehensively address determinants, strategies, and outcomes [45]. Implementation determinants identified in our interviews were organized using the CFIR, as specified a priori in our study design. Post hoc, we mapped implementation strategies for CDS that could enhance the implementation and sustainability of CDS, identified from the interview and ranking exercises, onto the Expert Recommendations for Implementing Change (ERIC) taxonomy [46]. Lastly, we mapped implementation outcomes for future measurement onto the Proctor outcomes framework, including those specified a priori in our survey instruments and additional outcomes highlighted in participant-derived data [33].

## Results

### Quantitative analysis

Demographic characteristics of participants (*n* = 26) are displayed in Table 1. Most participants were white cisgender female physicians. Almost all had heard of PrEP and over one-third self-reported prescribing PrEP. Providers rated the potential use of EHR-based CDS tools to improve HIV testing and PrEP delivery as acceptable (median score 5, IQR [4-5]) and appropriate (5, IQR [4-5]). However, feasibility ratings were slightly lower (4, IQR [3.75–4.75]), with 26% disagreeing that implementation would be “easy to do.” Of the seven proposed CDS tools (Table 2), the highest-ranking was the patient electronic self-screening tool, followed by alerts to providers for HIV screening for patients with STIs. The lowest overall ranking tool was default HIV testing orders. Rankings of CDS tools by ranged from 1 (highest) to 7 (lowest) for each of the tools.

**Table 1:** Demographics and provider experience in adolescent primary and sexual health care

**Provider characteristics (*n* = 26)**	*n* (%) [Median (IQR)]
**Age (years)**	46 (38–56)
**Race**	
White	24 (92)
Black/African American	1 (4)
Declined to answer	1 (4)
**Ethnicity**	
Hispanic or Latino	1 (4)
Non-Hispanic or Latino	25 (96)
**Sex**	
Male	3 (12)
Female	23 (89)
**Gender**	
Male	3 (12)
Female	23 (89)
**Primary practice location**	
Government-subsidized (“Title X”) Clinic	15 (58)
Non-Title X clinic	11 (42)
**Years in practice**	[18.5 (8–25)]
**Clinical degree**	
Doctor of Medicine/Doctor of Osteopathic Medicine	19 (73)
Certified Registered Nurse Practitioner	7 (27)
**Adolescent medicine training**	
Yes	7 (27)
No	19 (73)
**Number of adolescent patients seen per month**	[40 (15–60)]
**Frequency of documentation of sexual history in EHR**	
Infrequently or never	1 (4)
About half of the time	2 (8)
Most or all of the time	23 (89)
**Prior knowledge of HIV pre-exposure prophylaxis (PrEP)**	
Yes	23 (89)
No	3 (12)
**Prior experience prescribing PrEP**	
Yes	10 (39)
No	16 (62)
**Prior experience referring patients to another clinical site for HIV PrEP**	
Yes	13 (50)
No	13 (50)

**Table 2:** Ranking of electronic health record (EHR)-based CDS options for HIV/STI prevention care^a^

CDS Tool	Median ranking (IQR)
Electronic questionnaire for adolescents that populates sexual activity data into EHR^b^	2 (1–4.5)
Electronic alert to test for HIV for patients with STI diagnoses	4 (2–5)
Automated referrals to HIV prevention counselors for patients with STIs	4 (2–6)
Electronic alert to test for HIV in all adolescents	4 (2–6)
A bundled tool with both alerts and templated HIV test orders	4 (2–6)
An alert to counsel regarding PrEP for youth with STIs	4 (3–6)
Default HIV testing orders within the adolescent visit template (providers must uncheck to cancel order)	5 (3–6.25)

^a^Ranking from 1 to 7, where 1 is highest rank and 7 is lowest rank

^b^Missing *n* = 1

### Qualitative analysis

**Fig. 1:**
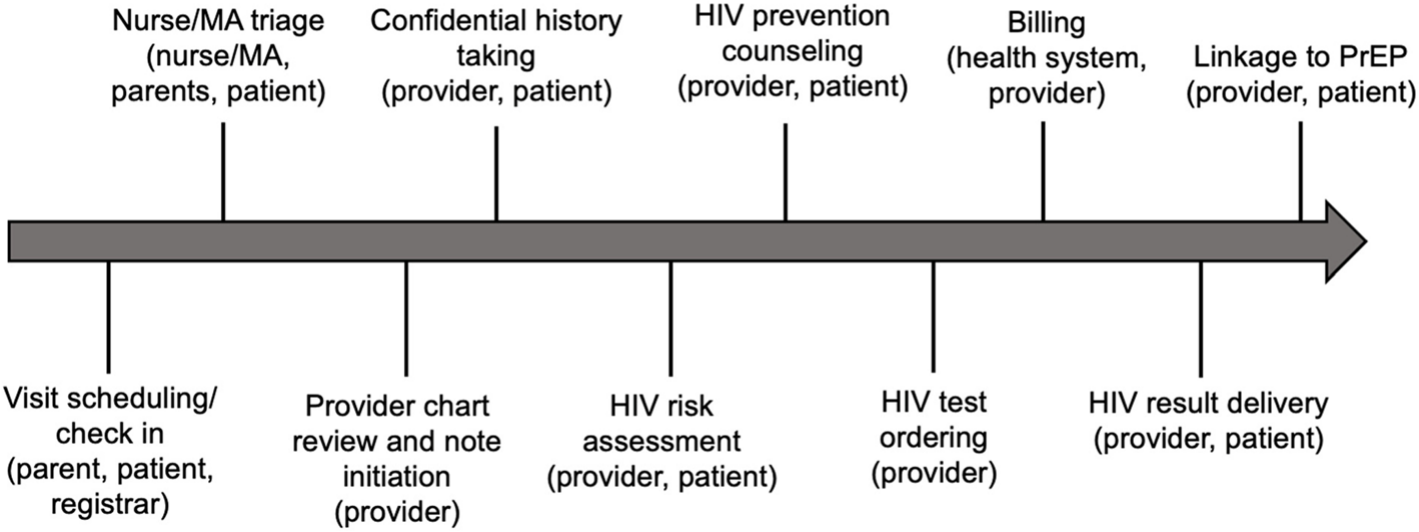
Work domain analysis: Visit workflow and key actors for HIV prevention at preventative care visits

We identified 10 unique decision-making steps of the pediatric well visit in the work domain analysis (Fig. 1). Key themes describing contextual barriers and facilitators of implementing HIV prevention CDS in pediatric visits are described below with exemplar quotes displayed in Table 3.

**Table 3:** Exemplar quotes by theme

Theme	Exemplar quotes
**Theme 1: Optimizing CDS integration within the primary care workflow (CFIR domain: Intervention)**	“I think having something come up for HIV testing if their chlamydia is positive isn’t as helpful because by then the patient’s out of the office, so I think if we’re going to be ordering it, it makes more sense to order it at the time of the visit regardless of what the results of the [STI test] are.”—General pediatrician, cisgender female
	“…I think, you’d find all the people onboard. I think, as I said, just please pilot things. Because we always start getting told that things are getting piloted, but sometimes they just get introduced. And then, having like…someone who doesn’t do this all the time onboard to kind of give feedback…”—General pediatrician, cisgender female
	“It comes from feeling like all we do is click now. It comes from feeling like we can’t – we’re not talking to our patients, we’re clicking boxes….”—General pediatrician, cisgender female
**Theme 2: Designing for standardization, with room for adaptable CDS design (Intervention)**	“I would strongly suggest in areas where chlamydia prevalence is greater than 20 percent that we engage in universal screening, for at least GC and chlamydia, and then maybe starting HIV at 15, doing it with rapid HIV, removing stigma, having everyone screened before they even see their provider, to remove bias.”—General pediatrician, cisgender female
	“If it’s a negative screen it’ll say negative screen. If it’s a positive, then it’ll give you suggestions and links on things that you can do. And if it’s emergent it’ll also save that for you. So, you want the tool to kind of not just ask the questions but help guide you through what to do with the answers.”—Adolescent Medicine physician, cisgender female
**Theme 3; Inner Setting (III) Recognizing the need for confidential care (Outer Setting)**	“…I feel like it’s possible that a female patient with private insurance might be more inclined to not want their parents to know – which would decrease testing.”—General pediatrician, cisgender female
**Theme 4: Improving accurate HIV risk perception (Outer Setting, Individuals)**	“I think females just don’t perceive that they’re as much at risk [of HIV infection]…adolescents just have a very skewed view of risk in general.”—General pediatrician, cisgender female
	“I think education…about numbers of people that are HIV positive in proportion of males versus females would be helpful…maybe looking at the data and seeing who's being tested and seeing where we miss might help us realize that we should be testing more than we are – so doing some data analysis and looking at the percent of males versus females that I test personally may really hit at home for me that I'm falling short even though right now I feel like I test as many, but I might not be.”—General pediatrician, cisgender female
**Theme 5: Recognizing limitations of time and staff as available resources (Inner Setting)**	“I know one of our nurses at [clinic] has been thinking about sort of like a much more nurse-led visit. And I think that could be something that allows you to better use all of your team members and take advantage of the time that the families and the – like less wasted time with them sitting around and more time when people are really working with them to address issues and prioritize.”—General pediatrician, cisgender female
**Theme 6: Prioritizing HIV relative to other primary care needs (Inner Setting)**	“I think, again, importance in priorities, and a lot of things are happening at that [visit], and you’re dealing with depression and school issues, and so [providers] have to pick and choose.”—Adolescent Medicine boarded pediatrician, cisgender female
	“I don't think people here deliberately ignore PrEP. I think in the grand scheme of everything that you have to deal with, with a teenager and you’re given 15, maybe 30 min at best… If you want to talk about every single thing that you could possibly talk about that this kid needs, you're well over an hour visit.”—Nurse practitioner, female
**Theme 7: Increasing provider knowledge about HIV testing and PrEP (Individuals)**	“So, I have never prescribed PrEP, so I wouldn't feel 100 percent comfortable prescribing it. But I would feel comfortable saying that she should consider that and recommend it.”—General pediatrician, cisgender female

#### Theme 1: Optimizing CDS integration within the primary care workflow (CFIR domain: Intervention)

Providers stressed the importance of placing CDS within the “right step” of the visit workflow, with particular consideration given to efficiency and confidentiality. For example, one provider noted that an EHR alert for PrEP counseling should not be triggered when parents are in the room due to risk of breaching confidentiality. Another consideration was a desire for CDS to be deployed while a patient is still physically in the clinic, to ensure CDS could be acted upon immediately, as opposed to during EHR chart documentation which is often asynchronous with the visit. Participants also wanted any new CDS to be piloted prior to implementation, recalling negative experiences with previous primary care EHR-based CDS that had not been piloted and were unsuitable for their workflow. Lastly, providers identified the need to consider the presence of other CDS in the workflow; too many unrelated CDS systems could contribute to a sense of burnout. While some providers highlighted successful changes in their own practice stemming from existing immunization and asthma-related CDS, providers also noted feeling overwhelmed by the sheer volume of EHR alerts and nudges. They raised concerns about additional CDS increasing the potential for alert fatigue—desensitization to EHR alerts due to an overwhelming number of alerts.

#### Theme 2: Designing for standardization, with room for adaptable CDS design (Intervention)

Providers raised the need for HIV screening CDS to be standardized for all adolescents, irrespective of HIV risk factors. Universal application of CDS could eliminate risk-based decision-making calculations using sexual history and lab data, thus reducing cognitive load. Universal CDS was also noted to have the potential to minimize provider implicit bias. Bias was most frequently discussed in the context of providers reporting being less likely to order HIV screening or consider PrEP for cisgender heterosexual female patients, as they were perceived as lower risk compared to sexual and gender minority youth. Finally, providers felt that standardization through universal alerts would increase fidelity to clinical guidelines and reduce stigma. As a complementary approach, in addition to universal CDS, other providers desired CDS elements that could deliver a broader scope of recommended resources and clinical guidance based on individual HIV risk. These providers desired algorithmic approaches using EHR data, such as STI results, to further customize decision support. In this approach, the format or type of CDS (for example, a simple pop-up alert to test for HIV for all patients versus links to a templated PrEP order set with CDC guidelines for those with recent STIs) could use objective data to provide more services to patients with higher vulnerability to HIV.

#### Theme 3: Recognizing the need for confidential care (Outer Setting)

Similar to the ubiquitous barrier of time pressure, the need to protect confidential sexual health information was a consideration at each workflow step. Participants noted that CDS alone would be unlikely to yield gains in HIV prevention in the absence of changes to funding and insurance billing practices to protect minor confidentiality. Specifically, providers discussed the utility of increasing access to federal Title X funding to allow expansion of confidential sexual health services. Providers had concerns about threats to confidentiality for privately insured patients due to explanations of benefits for HIV testing or PrEP services being sent to home addresses, risking disclosure of sexual activity and/or sexual orientation to parents. Some providers felt confidentiality concerns were more salient for cisgender female patients given norms by which female adolescents were perceived to experience more stigma around sexual activity than male adolescents.

#### Theme 4: Improving accurate HIV risk perception (Outer Setting, Individuals)

Many providers noted that adolescents, particularly those with STIs, did not perceive themselves as at-risk for HIV or eligible for PrEP. This mismatched risk perception was seen as a barrier to HIV testing and PrEP uptake that would not be addressed by CDS, suggesting that additional patient-facing interventions were needed to increase HIV testing and PrEP uptake. In addition to adolescent patients’ self-assessment of HIV risk, providers’ perceptions of patient risk also influenced their clinical decision-making. Some providers expressed that CDS focused on universal HIV testing could be a waste of resources, perceiving that cisgender female adolescents are at very low risk of acquiring HIV.

#### Theme 5: Recognizing limitations of time and staff as available resources (Inner Setting)

Providers emphasized that CDS development should consider clinic time as a critical resource, given the extensive array of clinical responsibilities at well visits. Providers also expressed the need for additional resources to maximize CDS effectiveness, such as rapid HIV tests, implicit bias training for providers, and patient education materials. Providers noted that time challenges were often greatest for cisgender female patients. Given the need to also discuss menstruation and contraception during sexual health conversations, competing priorities diminished time for addressing HIV testing and PrEP counseling. Participants also suggested that CDS alone would not improve HIV prevention without changes to staff roles and workflow. Providers stressed the need for involvement of staff other than pediatricians to optimize HIV testing and prevention counseling. Two examples—medical assistant-led HIV rapid testing and nurse-led HIV prevention counseling—involved shifting responsibilities of pre-test counseling and result delivery to allied members of the healthcare team.

#### Theme 6: Prioritizing HIV relative to other primary care needs (Inner Setting)

Many providers felt that while HIV and PrEP were important topics, they fell below more prevalent and immediate health concerns, such as depression, school performance, and obesity. Providers also reported concerns about having insufficient time to address HIV given priority placed on mandated health system performance measures, including depression screening. Perceptions of the relative priority of HIV also influenced providers’ attitudes toward implementing HIV prevention CDS. Some providers had concerns about feeling “forced” to discuss PrEP without having sufficient knowledge or time to do so. However, others noted that their clinic would and should be prepared to talk about HIV prevention and PrEP as part of their purview as primary care providers.

#### Theme 7: Increasing provider knowledge about HIV testing and PrEP (Individuals)

Providers noted that their knowledge (or lack thereof) regarding HIV testing and PrEP would influence how effectively they could utilize HIV prevention CDS. For example, for providers with no prior knowledge of PrEP, CDS would need to efficiently provide education, links to HIV prevention guidelines, or be accompanied by educational efforts to increase competency across clinical staff.

### Integration of findings

**Fig. 2:**
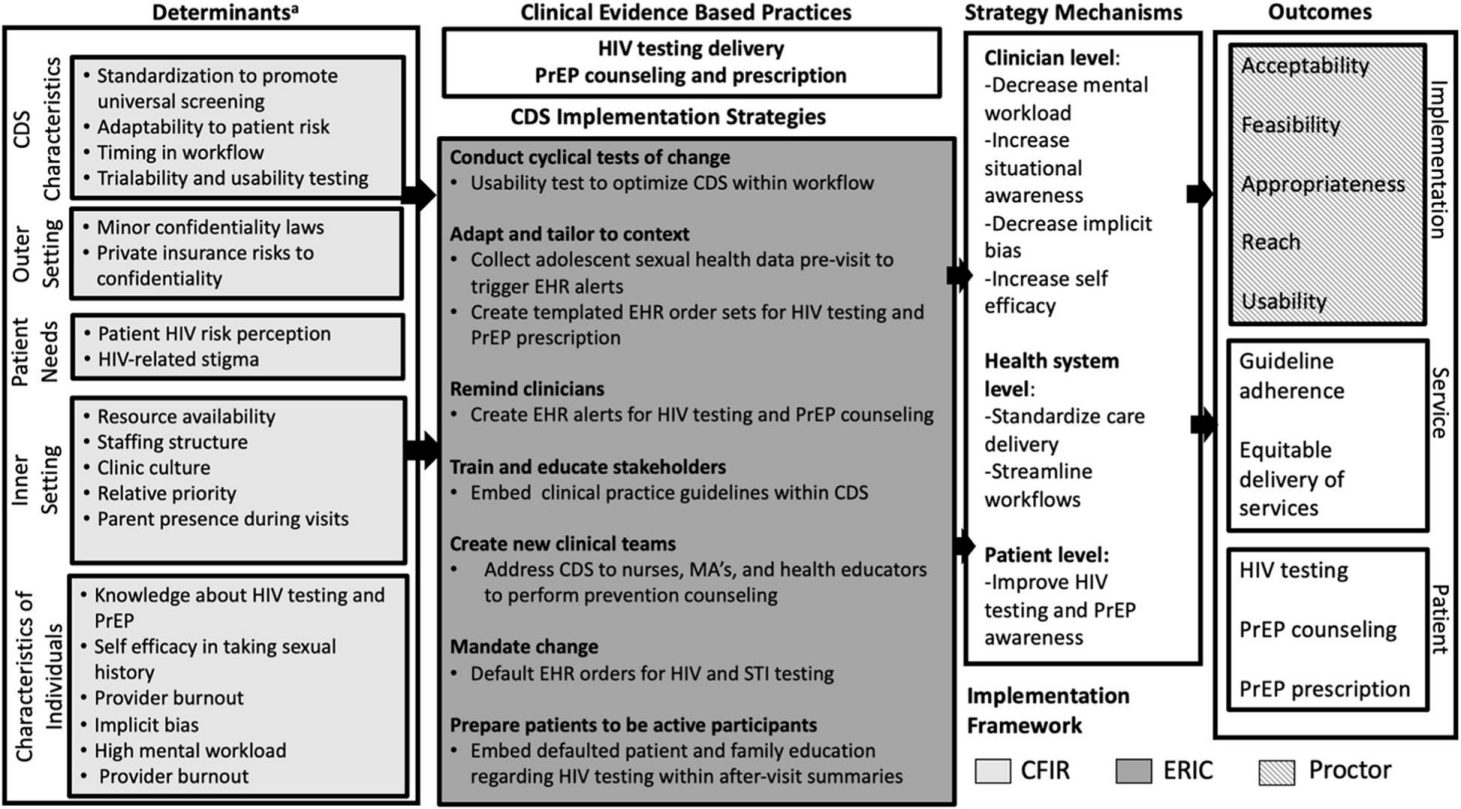
Implementation Research Logic Model: electronic clinical decision support to improve HIV testing and PrEP delivery

In Fig. 2, we synthesize our key findings and provide an Implementation Research Logic Model for CDS implementation for HIV prevention-oriented pediatric primary care. Within the model, we propose seven essential strategies for CDS implementation framed within the ERIC taxonomy that are based on participant survey responses and the qualitative themes described above: (1) Conducting cyclical usability testing to optimize CDS within the workflow, (2) adapting to context by using pre-visit confidential patient-collected sexual health information to trigger CDS early in the visit, (3) providing strong guidance reinforcing equity by introducing defaulted HIV testing orders to improve universal testing, (4) using EHR alerts to remind providers of need for HIV prevention within busy clinical care encounters, (5) embedding clinical practice guidelines within CDS to increase provider knowledge, (6) expanding clinical teams to shift HIV prevention responsibilities away from pediatricians and advanced practice providers and (7) educating patients to increase awareness of their need for HIV testing.

## Discussion

In this pre-implementation study, we performed a deep contextual inquiry and developed an Implementation Research Logic Model to improve the reach and equity of HIV testing and PrEP delivery at adolescent preventative care visits. With respect to determinants of CDS implementation, in our logic model (light gray boxes, Fig. 2) limited time to provide HIV prevention care at primary care visits was one of the most salient themes in both the work domain and CFIR-based analyses. The American Academy of Pediatrics (AAP) Bright Futures framework recommends five mandated health screenings, seven risk-based screenings, and nineteen potential topics for anticipatory guidance at the 15–17-year-old well visit [47]. Exploring how interventions are deployed within existing workflows is consistent with best practices for improving CDS usability as delineated by the U.S. Department of Health and Human Services Agency for Healthcare Research and Quality and previous analyses [28, 48–50]. Our findings demonstrate the need for future CDS to address competing priorities and providers’ high mental workload within primary care visits. By using work domain analysis, we identified key implementation considerations, such as the critical need to maintain patient confidentiality, within the unique workflow of an adolescent well visit.

The time pressures described by participants also reinforced the potential for implicit bias to impact perceptions of patients’ need or eligibility for HIV testing and PrEP services. Participants discussed their own and colleagues’ biases wherein cisgender heterosexual females were perceived of being at very low risk for HIV infection, and thus often not screened for HIV or considered for PrEP. These findings suggest that providers may narrow in on a patient’s sexual orientation, sex, or gender, at the expense of objective biomarkers that indicate increased HIV risk, such as STIs or evidence-based recommendations for universal testing irrespective of HIV risk. While cisgender adolescent girls account for only 12% of U.S. youth HIV diagnoses, these data need to be contextualized in the very low HIV screening rates in this population. Only 9% of high school students have ever received an HIV test, and nearly half of youth with HIV have not been tested and are therefore unaware of their diagnosis [51]. Additionally, PrEP awareness and adoption remain low among adolescent MSM and transgender youth, despite recognition of the importance of PrEP for these key populations [52, 53].

Based on our interview data, we added equity as an additional Implementation Outcome in our logic model (Fig. 2, striped box). Future CDS interventions should carefully measure not only gains in HIV testing and PrEP delivery, but also equitable delivery across populations of adolescents.

With respect to implementation strategies (Fig. 2, dark gray box), most providers noted the importance of CDS supporting universal screening. In the context of AAP and CDC recommendations for universal HIV screening, and recent CDC guidance that all sexually active individuals receive information about PrEP [8], our results support designing CDS which provides a universal prompt for HIV screening for all adolescents to maximize reach and increase equity [9]. However, our qualitative and quantitative findings also demonstrate high appeal for the implementation strategy of adapting the CDS to a patient’s unique context. In this strategy, pre-visit patient self-reported data on sexual activity, sexual orientation, and gender identity, combined with STI lab test data, could trigger a stronger prompt to not only test for HIV, but to also counsel on PrEP or provide additional prevention services. Prior studies have shown success with electronic collection of pre-visit data, including patient sexual orientation and gender identity information [54, 55]. Having sexual activity data available at the start of the visit decreases reliance on provider self-efficacy to discuss sexual health and avoids biases by circumventing provider judgments on “who to screen” for sexual activity. Including sexual orientation and gender identity data can also allow for culturally humble tailoring of sexual health information for sexual and gender minority adolescents.

While CDS was the primary focus of our research, clinicians noted that attending to structural issues within both the inner and outer settings, including availability of rapid testing, staffing structures, and assuring confidentiality of care within the clinic and throughout billing processes, are essential for improving HIV prevention care delivery in pediatric primary care [56–59]. Notably, providers identified task-shifting among the healthcare team as one structural area to explore. Restructuring workflows, including shifting HIV testing and PrEP counseling to nurses or health educators, has been demonstrated to be a promising strategy for improving HIV prevention service delivery [56, 57, 60]. From a CDS perspective, task-shifting also provides opportunities to have multiple target users for CDS including nurses, medical assistants, and front-desk staff.

Our study has several limitations. Although our sample was similar in characteristics to the network primary care provider population, which is composed of 83% female and 84% physician providers, most participants were white female physicians, which may limit generalizability. We do not have data on providers who opted not to participate in the study, whose perspectives may differ from participants’. Our data were derived from a large urban health system in a high HIV prevalence region and participants may therefore have higher levels of PrEP awareness than typical. However, participants ranged from those who felt confident prescribing PrEP to those who had never heard of PrEP. While participants frequently noted high mental workload as a barrier to HIV testing and PrEP delivery, our study did not include formal measures of mental workload, many of which require complex physiologic measurement [61]. There is a need for both development of pragmatic measurement strategies for mental workload, and inclusion of these measures in future studies. Participants did not receive interview transcripts or provide feedback on study findings. Future studies will benefit from triangulation of findings with provider teams. We provided example CDS tools and content to participants in the ranking exercise completed prior to the interview, which may have affected providers’ discussions regarding CDS tools, barriers, and facilitators. However, these examples were based on existing health system CDS with which participants were already familiar. Lastly, given our small sample size and high level of acceptability of a proposed CDS system, we were unable to compare themes between providers by attitudes toward using CDS to improve HIV prevention delivery. However, we were able to achieve saturation of themes across the interviews.

## Conclusions

Our study provides a detailed understanding of the factors that providers view as most influential in the use of CDS to improve equitable delivery of HIV testing and PrEP services in adolescent primary care. For future implementation efforts, our findings suggest that prioritizing CDS system development early in the visit, focused on pre-visit self-reported risk screening to drive alerts, may have the greatest downstream yields in increasing efficiency and decreasing cognitive burden. Furthermore, future CDS interventions should address the themes that were most prominent across all visit steps: time and staffing constraints, confidentiality of sexual health information, and provider and patient knowledge and self-efficacy. By contextually focusing CDS efforts on the specific needs of the pediatric primary care environment, health systems can maximize effectiveness gains in improving equitable delivery of HIV prevention services.

## Supplementary Information

**Additional file 1. **Consolidated criteria for reporting qualitative research (COREQ): a 32-item checklist for interviews and focus groups.

**Additional file 2: Supplementary Figure 1.** Interview Vignettes.

## Data Availability

Data may be made available through formal data sharing agreement with the authors’ institution.

## Abbreviations

AAP: American Academy of Pediatrics
AIM: Acceptability of Intervention Measure
CDC: Centers for Disease Control and Prevention
CDS: Clinical decision support
CFIR: Consolidated Framework for Implementation Research
EBPs: Evidence-based practices
EHR: Electronic health record
ERIC: Expert Recommendations for Implementing Change
FIM: Feasibility of Intervention Measure
IRLM: Implementation Research Logic Model
IAM: Intervention Appropriateness Measure
MSM: Men who have sex with men
PrEP: Pre-exposure prophylaxis
STI: Sexually transmitted infection
