# Supplementary material for: Provider perspectives on clinical decision support to improve HIV prevention in pediatric primary care: a multiple methods study
Source: Implement Sci Commun. 2023 Feb 21;4:18. doi: 10.1186/s43058-023-00394-7 (PMC9945664; doi:10.1186/s43058-023-00394-7)
Supplement: Supplementary file 1 — Additional file 1. Consolidated criteria for reporting qualitative research (COREQ): a 32-item checklist for interviews and focus groups. [file 43058_2023_394_MOESM1_ESM.docx]

**Consolidated criteria for reporting qualitative research (COREQ): a 32-item checklist for interviews and focus groups**

| No | Item | Guide questions/description | Section |
| --- | --- | --- | --- |
| **Domain 1: Research team and reflexivity**  Personal Characteristics | | | |
| 1 | Interviewer/facilitator | Which author/s conducted the interview or focus group? | Methods: Study Procedures |
| 2 | Credentials | What were the researcher’s credentials? *E.g. PhD, MD* | Methods: Study Procedures |
| 3 | Occupation | What was their occupation at the time of the study? | Methods: Study Procedures |
| 4 | Gender | Was the researcher male or female? | Methods: Study Procedures |
| 5 | Experience and training | What experience or training did the researcher have? | Methods: Study Procedures |
| Relationship with participants | | | |
| 6 | Relationship established | Was a relationship established prior to study commencement? | Methods: Study Procedures |
| 7 | Participant knowledge of the interviewer | What did the participants know about the researcher? *e.g. personal goals, reasons for doing the research* | Methods: Design, Setting, and Participants |
| 8 | Interviewer characteristics | What characteristics were reported about the interviewer/facilitator? *e.g. Bias, assumptions, reasons and interests in the research topic* | Methods: Design, Setting, and Participants |
| **Domain 2: study design**  Theoretical framework | | | |
| 9 | Methodological orientation and theory | What methodological orientation was stated to underpin the study? *e.g. grounded theory, discourse analysis, ethnography, phenomenology, content analysis* | Methods: Qualitative Measures |
| Participant selection | | | |
| 10 | Sampling | How were participants selected? *e.g. purposive, convenience, consecutive, snowball* | Methods: Design, Setting, and Participants |
| 11 | Method of approach | How were participants approached? *e.g. face-to-face, telephone, mail, email* | Methods; Design, Setting, and Participants |
| 12 | Sample size | How many participants were in the study? | Results: Quantitative Analysis; Table 1 |
| 13 | Non-participation | How many people refused to participate or dropped out? Reasons? | Methods: Design, Setting, and Participants |
| Setting | | | |
| 14 | Setting of data collection | Where was the data collected? *e.g. home, clinic, workplace* | Methods: Study Procedures |
| 15 | Presence of non-participants | Was anyone else present besides the participants and researchers? | Methods: Study Procedures |
| 16 | Description of sample | What are the important characteristics of the sample? *e.g. demographic data, date* | Results: Quantitative Analysis; Table 1 |
| Data collection | | | |
| 17 | Interview guide | Were questions, prompts, guides provided by the authors? Was it pilot tested? | Methods: Qualitative Measures |
| 18 | Repeat interviews | Were repeat interviews carried out? If yes, how many? | Methods: Study Procedures |
| 19 | Audio/visual recording | Did the research use audio or visual recording to collect the data? | Methods: Study Procedures |
| 20 | Field notes | Were field notes made during and/or after the interview or focus group? | Methods: Study Procedures |
| 21 | Duration | What was the duration of the interviews or focus group? | Methods: Study Procedures |
| 22 | Data saturation | Was data saturation discussed? | Methods: Design, Setting, and Participants |
| 23 | Transcripts returned | Were transcripts returned to participants for comment and/or correction? | Discussion |
| **Domain 3: analysis and findings**  Data analysis | | | |
| 24 | Number of data coders | How many data coders coded the data? | Methods: Qualitative Analysis |
| 25 | Description of the coding tree | Did authors provide a description of the coding tree? | Methods: Qualitative Analysis |
| 26 | Derivation of themes | Were themes identified in advance or derived from the data? | Methods: Qualitative Analysis |
| 27 | Software | What software, if applicable, was used to manage the data? | Methods: Qualitative Analysis |
| 28 | Participant checking | Did participants provide feedback on the findings? | Methods: Study Procedures |
| Reporting | | |  |
| 29 | Quotations presented | Were participant quotations presented to illustrate the themes / findings? Was each quotation identified? *e.g. participant number* | Table 3; N/A |
| 30 | Data and findings consistent | Was there consistency between the data presented and the findings? | Results |
| 31 | Clarity of major themes | Were major themes clearly presented in the findings? | Results: Qualitative Analysis |
| 32 | Clarity of minor themes | Is there a description of diverse cases or discussion of minor themes? | Results: Qualitative Analysis |

Tong A, Sainsbury P, Craig J. Consolidated criteria for reporting qualitative research (COREQ): a 32-item checklist for interviews and focus groups. Int J Qual Health Care. 2007 Dec;19(6):349-57.
