# Supplementary material for: Provider perspectives on clinical decision support to improve HIV prevention in pediatric primary care: a multiple methods study
Source: Implement Sci Commun. 2023 Feb 21;4:18. doi: 10.1186/s43058-023-00394-7 (PMC9945664; doi:10.1186/s43058-023-00394-7)
Supplement: Supplementary file 2 — Additional file 2: Supplementary Figure 1. Interview Vignettes. [file 43058_2023_394_MOESM2_ESM.docx]

**Supplementary Figure 1: Interview Vignettes**

Vignette #1: You are seeing a 17 year-old girl for her well child visit. She has a history of asthma and seasonal allergies. On social history, she has had one sexual partner in the past year. Her PHQ-9 score identifies her as depressed with passive suicidal ideation. You assess her labs in EPIC chart review and note that she had gonorrhea six months ago. In addition to referring her to social work, you order a urine aptima for her.

Vignette #2: You are seeing a 15 year-old male who has sex with men for a sick visit for STI treatment. He tested positive for gonorrhea earlier this week at his well visit. He was last tested for HIV three months ago, and his test was nonreactive.

Vignette #3: You are seeing an 18 year-old female for STI treatment. She tested positive for gonorrhea two days ago and is coming in for STI treatment. She has never had an HIV test.

1. In this scenario, what other information would you look for in the chart to support decision making?
2. Can you walk us through how you would complete HIV testing? What might be some of the barriers to completing HIV testing? What are some things that may make it easier?
3. What additional counseling would you want to offer this patient? Since PrEP may be recommended for this patient would you feel comfortable counseling him/her about HIV prevention medication? What are some of the barriers?

Prompts for all vignettes:

- How would the visit flow differently if this was a long-time, vs new patient?
- How might you use family planning resources in this scenario?
- How might the visit flow differently if a parent were present?
